# High-resolution rectoscopy using MHz optical coherence tomography: a step towards real time 3D endoscopy

**DOI:** 10.1038/s41598-024-55338-5

**Published:** 2024-02-26

**Authors:** Berenice Schulte, Madita Göb, Awanish Pratap Singh, Simon Lotz, Wolfgang Draxinger, Marvin Heimke, Mario pieper, Tillmann Heinze, Thilo Wedel, Maik Rahlves, Robert Huber, Mark Ellrichmann

**Affiliations:** 1 Interdisciplinary Endoscopy, Medical Department 1, University Hospital Schleswig-Holstein, Campus Kiel, Kiel, Germany; 2 Institute of Biomedical Optics, University of Luebeck, Luebeck, Germany; 3 Center of Clinical Anatomy, Institute of Anatomy, Christian-Albrechts University Kiel, Kiel, Germany; 4 Institute of Anatomy, University of Luebeck, Luebeck, Germany; 5 Airway Research Center North (ARCN), German Center for Lung Research (DZL), Luebeck, Germany

**Keywords:** Anatomy, Gastroenterology, Optics and photonics

## Abstract

Colonoscopy and endoscopic ultrasound play pivotal roles in the assessment of rectal diseases, especially rectal cancer and inflammatory bowel diseases. Optical coherence tomography (OCT) offers a superior depth resolution, which is a critical factor for individualizing the therapeutic concept and evaluating the therapy response. We developed two distinct rectoscope prototypes, which were integrated into a 1300 nm MHz-OCT system constructed at our facility. The rapid rotation of the distal scanning probe at 40,000 revolutions per minute facilitates a 667 Hz OCT frame rate, enabling real-time endoscopic imaging of large areas. The performance of these OCT-rectoscopes was assessed in an ex vivo porcine colon and a post mortem human in-situ colon. The OCT-rectoscope consistently distinguished various layers of the intestinal wall, identified gut-associated lymphatic tissue, and visualized a rectal polyp during the imaging procedure with 3D-reconstruction in real time. Subsequent histological examination confirmed these findings. The body donor was preserved using an ethanol-glycerol-lysoformin-based technique for true-to-life tissue consistency. We could demonstrate that the novel MHZ-OCT-rectoscope effectively discriminates rectal wall layers and crucial tissue characteristics in a post mortem human colon in-situ. This real-time-3D-OCT holds promise as a valuable future diagnostic tool for assessing disease state and therapy response on-site in rectal diseases.

## Introduction

In the era of personalized therapeutic strategies for rectal diseases, including rectal cancer and inflammatory bowel disease (IBD), there is a critical need for precise diagnostic methods and advancements in high-resolution, in vivo phentotyping by endoscopic imaging techniques. Conventional high-definition, white-light colonoscopy, only assessing the mucosal surface, exhibits a significant adenoma miss rate in colorectal cancer screening and substantial interobserver variabilities in evaluating inflammation among patients with IBD^1^. Especially in IBD, transmural healing has emerged as a crucial therapeutic target. Unlike the conventional treatment, which focuses on achieving symptomatic relief and mucosal healing, transmural healing aims to restore health of all layers of the intestinal wall^2^. This approach is pivotal in preventing disease relapse, reducing complications, and enhancing the long-term outcomes and quality of life for patients.

In the domain of rectal cancer, endoscopy plays a vital role in the initial tumor staging and in assessing the response to neoadjuvant chemo-radiotherapy. However, accurately determining a complete clinical response following neoadjuvant therapy is fraught with challenges^3^. Therapy-induced changes such as inflammation, scarring, or fibrosis can lead to potential false positives, while residual transmural disease might result in false negatives. Furthermore, the variability in interpreting endoscopic findings among clinicians introduces a degree of subjectivity and interobserver variability, highlighting the increasing demand for more precise and standardized criteria, along with advanced imaging modalities, to evaluate complete clinical response^4^.

In addressing these challenges, rectal endoscopic ultrasound (EUS) emerges as a valuable modality for visualizing distinct wall layers and extraluminal, adjacent tissue structures. It plays a critical role in rectal cancer imaging and allows for the quantification of inflammation levels in IBD by measuring the total wall thickness^5^. However, the resolving power of EUS falls short in distinguishing fine structures, emphasizing the need for technologies with more detailed tissue analysis. In response to these limitations, the introduction of Confocal Laser Endomicroscopy (CLE) signifies a substantial advancement in the assessment of superficial tissue structures. CLE, functioning as a probe-based endoscopic technique, facilitates in vivo microscopic imaging of the mucosal surface during routine endoscopic examinations^6^. Beyond defining suspicious lesions and facilitating targeted biopsies and resections in tumor diagnosis^7^, CLE also plays a role in indicating intestinal barrier healing in IBD^6^. However, the absence if sufficient depth resolution represents a crucial constraint in evaluating inflammation and staging of tumors.

Optical Coherence Tomography (OCT) emerges as a technical solution that integrates the advantages of both EUS and CLE, offering depth-dependent, high-resolution tissue visualization^8^. OCT offers the non-invasive acquisition of complete three-dimensional image datasets with microscopic precision, which has been explored in preliminary research. These study highlights its potential in gastroenterology, with encouraging endoscopic colon OCT images demonstrated in animal models^9–15^, as well as ex vivo in human samples^16–20^. In initial clinical attempts^16,21–26^, a probe-based OCT system was predominantly used, employing catheters licensed for coronary applications. Trindade et al. utilized a clinically lincensed volumetric OCT system (NvisionVLE; NinepointMedical, Bedford, MA, USA), which operated at a relatively modest pace, scanning a 6 cm circumferential segment in 90 s; however, it is no longer commercially available^18^.

Despite OCT's considerable advantages, its application in clinical settings remains an area of active investigation, largely due to the need for extremely high acquisition speeds to capture full three-dimensional data of large areas without motion artifacts^17,27^. The OCT imaging speed is constrained by the A-scan rate of the OCT system and the endoscope's scanning speed. However, the introduction of Fourier Domain Mode Locking (FDML) lasers has enabled unprecedented imaging speeds beyond several million A-scans per second: Megahertz-OCT (MHz-OCT)^28,29^. The high acquisition speed of FDML-based MHZ-OCT offers numerous advantages. It provides large fields of view (FOV) within a reasonable timeframe by scanning many tens of square centimeters in seconds^30^. Moreover, it enables real-time 4D OCT applications, wherein increased speed can augment image quality and introduce vital functional contrast. This is particularly crucial for interactive clinical procedures or surgical navigation^31,32^.

## Methods

### Study overview and ethics statement

As a first step towards achieving a full 3D OCT colonoscopy, we initiated the development of a rigid FDML-based MHz-OCT rectoscope This rectoscope incorporates an innovative low-cost drone motor design, specifically tailored to facilitate real-time 3D visualization of rectal diseases in a clinical setting. We conducted a methodical exploration through a series of imaging experiments to validate the applicability of our rectoscope. The exploration started with the use of a silicone colon model to establish baseline imaging benchmarks. Subsequently, we moved on to ex vivo porcine colonic tissue to address the complexities of biological imaging. Finally, we examined the intricate details of the human anatomy within a post mortem human colon specimen in situ. This progression not only tested the operational feasibility and performance of our MHz-OCT rectoscope across a spectrum of increasingly complex scenarios but also aimed to refine our understanding of its potential in real-world clinical settings. It is noteworthy that the technological and clinical aspects of this rectoscope possess the potential to impact a broader range of colorectal conditions.

The use of two probes was deemed necessary for this study due to the limitations of the initial probe in achieving the required lateral resolution and stability^33^, which were crucial for the accurate visualization of colonic structures. The implementation of a second-generation probe was found to significantly improve imaging accuracy and reduce overall jitter, resulting in clearer and more reliable OCT images^34^. The upgrade was complemented by benchmark comparisons using a benchtop MHz-OCT unit. This enabled us to showcase the high-resolution image capturing capabilities of our rectoscope, which are similar to those of leading OCT systems. By incorporating histological verification in our methodology, we have further strengthened the validity of our imaging results. This ensures that the OCT-derived visualizations accurately represent the histopathological architecture. Therefore, we believe that the findings of this study hold the potential to significantly advance gastrointestinal imaging practices.

The whole study concept was approved by the Internal Review Board of the Christian-Albrechts-University, Kiel, Germany (No. D420/22). The freshly explanted porcine colon was obtained from a local butcher, classified as slaughterhouse waste. The body donor was obtained from the body donation program of the Institute of Anatomy, Christian-Albrechts-University, Kiel, Germany. The body donation program was approved by the legal department, Christian-Albrechts-University Kiel, Germany (KS-2003). It is important to note that all body donors in this program have previously provided written informed consent for the utilization of their bodies in both medical research and educational purposes in accordance with the relevant guidelines and regulations.

All studies with post mortem animal or human biomaterial were conducted with strict adherence to established guidelines and regulations to ensure ethical and scientific compliance.

### Optical Coherence Tomography

**Figure 1:**
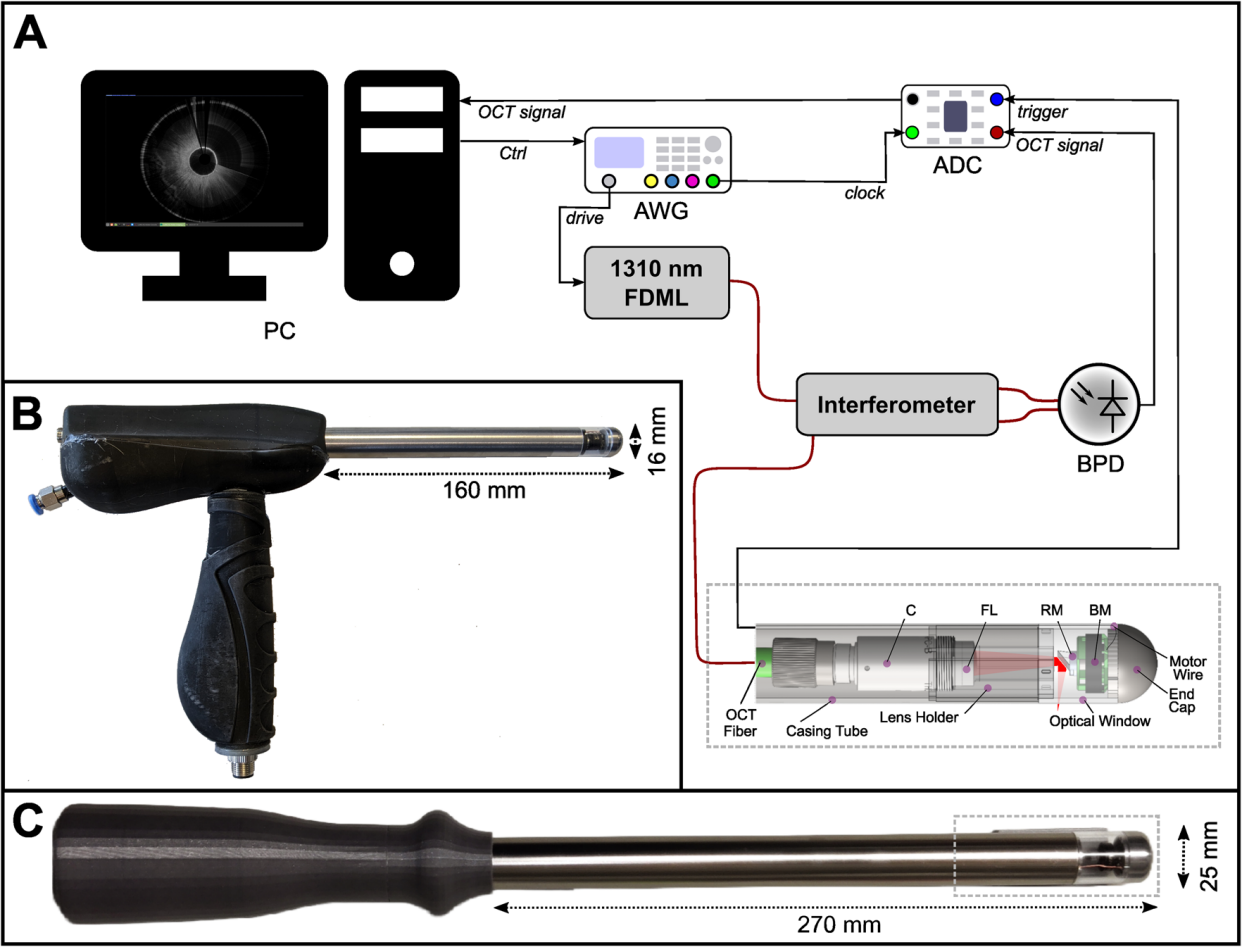
Experimental Setup. (**A**) MHZ-OCT rectoscope system with a schematic of the probe tip (dashed rectangle) corresponding to C. (**B**) 16 mm OCT rectoscope. (**C**) 25 mm OCT rectoscope. Abbreviations: ADC, analog to digital converter; AWG, arbitrary waveform generator; BM, brushless DC Motor; BPD, balanced photodetector; C, collimator; FDML, Fourier domain mode locking; FL, focusing lens; PC, Personal Computer; RM, reflective mirror; red wires: optical fiber.

In this study, we utilized a home-built FDML laser-based MHz-OCT system, which is similar to the one described by Göb et al.^35^. The FDML laser operates at a tuning-rate of 410 kHz with eight-times optical buffering, resulting in an effective A-scan rate of 3.3 MHz. The spectral bandwidth was set to 100–110 nm, which corresponds to an axial resolution of ~ 8 µm and an imaging range of ~ 5 mm in air. The OCT interference fringes were detected using a 1.6-GHz balanced photodetector (Thorlabs, PDB480C-AC, USA), and the output signal was acquired using a high-speed digitizer (AlazarTech, ATS9373, Canada) operating at a 12-bit sampling depth with a sampling rate of 4GS/s. A schematic setup of the OCT system with integrated rectoscope is displayed in Fig. 1A.

To achieve real-time, low-latency display for interactive positioning and aiming mode, we developed a specific software solution for handling OCT data. This real-time processing and visualization of OCT data are based on our custom in-house software designed for online graphical processing unit (GPU) accelerated OCT processing, referred to as “Luebeck University Real-time OCT” (LURO) navigation^31,32^. LURO enables the live display of the OCT rectoscope in Cartesian or polar coordinates. A detailed technical description is enclosed in the supplementary material.

### OCT Probes

The development of our OCT probes has been an iterative process, aimed at optimizing imaging quality and operational efficiency for intestinal imaging. Our initial efforts resulted in the prototype of a side-viewing rectoscope, detailed in Fig. 1B. This first version (RV1) featured a 16 mm diameter, incorporating a brushless DC motor with bush bearing (BETAFPV, 0703, China) for rotational scanning. The optical assembly comprised a fiber-collimator (Thorlabs, F260APC-C, USA) and a 20 mm focusing lens (Thorlabs, AC080-020-C, USA), which together achieved a spot size of 19 µm in air, despite a theoretical design targeting 12 µm^33^. This probe employed a Polymethyl methacrylate (PMMA) window with a 1 mm thickness for circumferential scanning, ensuring both strength and maintaining imaging clarity without compromise. The complete technical specifications and performance characteristics of this RV1 probe are detailed in the respective reference^33^.

Recognizing the limitations and potential areas for improvement observed with the first prototype, our development efforts led to the advent of a second-generation rectoscope (RV2), showcased in Fig. 1C. This refined model, with a 25 mm diameter, maintained the rotational speed of 40,000 rpm but featured substantial enhancements, including a more robust brushless DC motor with ball bearing (BETAFPV, 1102, China), a different fiber-collimator (Thorlabs, F240APC-C, USA), and a 30 mm focusing lens (Thorlabs, AC080-030-C, USA). These adjustments yielded a spot size of approximately 35 µm in air, closely matching the theoretical expectation of 33 µm. Due to an increased motor diameter, a PMMA window of 25 mm diameter and 2 mm thickness was used as outer shielding. Detailed information on the second-generation probe's design and its impact on imaging outcomes can be found in the respective reference^34^.

Both generations of probes used a reflective mirror (Thorlabs, RA03-M01, USA) angled at 5° to the motor axis to prevent undesirable reflections and enhance image quality. The rotational imaging unit is situated at the tip of a 27 cm long metal shaft, which is connected to a 3D-printed ergonomic handpiece that houses the electronic motor drive unit. To synchronize the motor speed with the OCT acquisition, the motor’s back electromotive force (BEMF) signal was used as OCT frame trigger. This virtual Hall sensing approach could reduce non-uniform rotational distortion (NURD) artifacts to less than 4 mrad^33^, which enabled quasi phase-stable OCT imaging and instantaneous real-time visualization without the need for post-processing corrections^33^. A rotation speed of 40,000 rpm was chosen to enable a frame rate of ~ 667 Hz in line with the acquisition rate of the OCT-system. We employed a manual pullback speed ranging between approximately 5–10 mm/s, considering the current spot size of 19 µm for RV1 and 35 µm for RV2 probes, respectively.

To further validate and refine our RV2 probe, we employed a benchtop OCT system as a complementary evaluation tool. This approach enabled a thorough comparison of imaging performance and operational efficiency, ensuring that our probes not only meet but exceed the requirements for advanced intestinal imaging applications. Table 1 summarizes the key performance metrics of both probes, offering a comprehensive overview of our technological advancements and their implications for clinical practice.

**Table 1:** Technical specifications of OCT probes detailing their operational parameters for intestinal imaging.

Probe	Diameter (mm)	B-scan rate (Hz)	Measured Lateral Resolution (μm)	Rayleigh range (μm)	Pullback Speed (mm/s)	Scan volume (voxels)	Scan time (s)	Jitter (A-Scan)	Technical reference
RV1	16	~ 667	19	85	~ 5	4800 × 1334 × 1200	2	± 40	Ref^33^
RV2	25	~ 667	35	665	~ 10	4900 × 5120 × 1200	7.67	± 4	Ref^33^

### Experimental imaging setups

#### Silicone colon model

**Figure 2:**
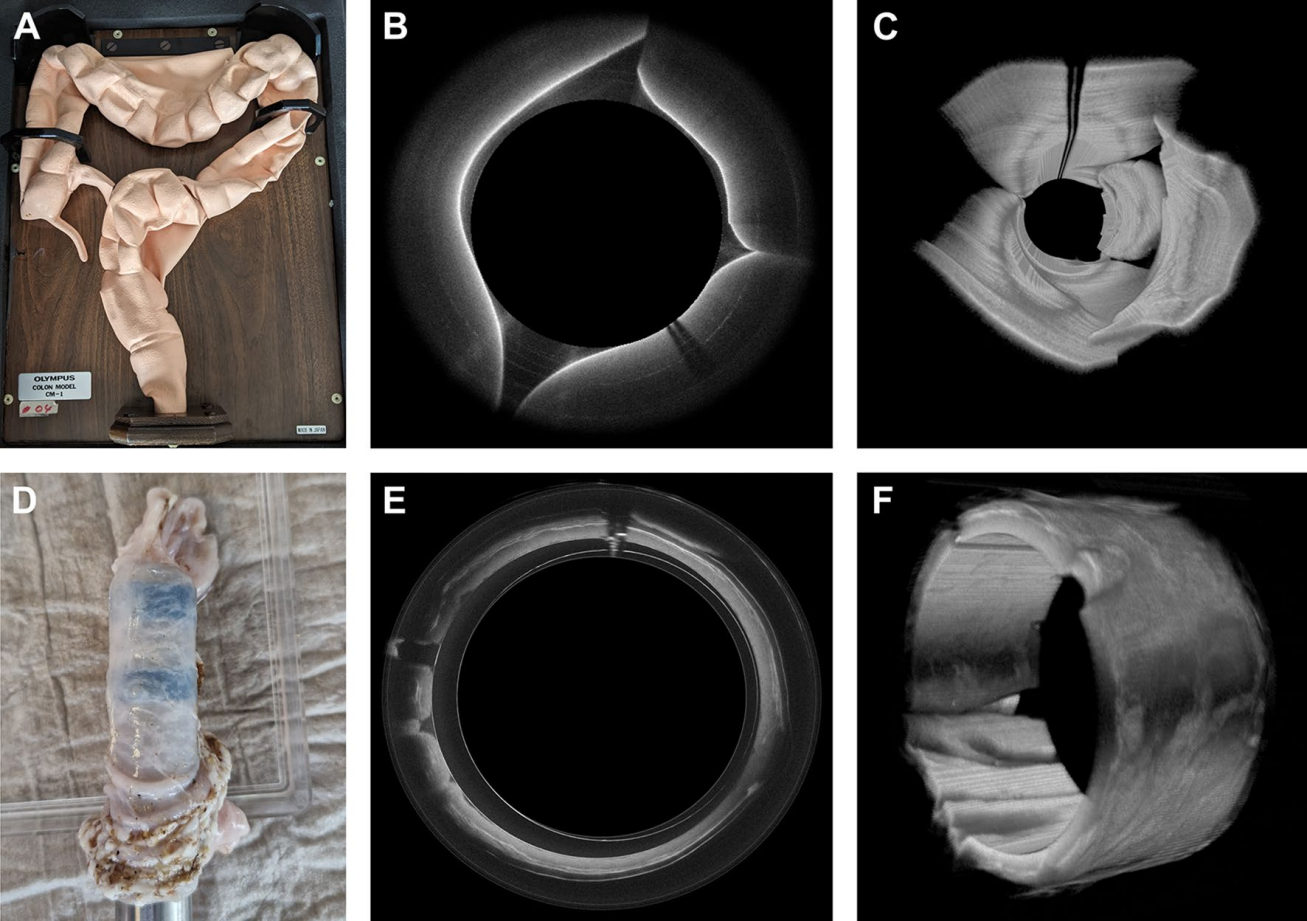
Endoscopic OCT of a silicon colon model and porcine colon. (**A**) Silicon model. (**B**) OCT B-scan of the silicon model (average of 10 consecutive frames, displayed in polar coordinates). (**C**) OCT 3D view of the silicon colon model. (**D**) OCT rectoscope inserted into a porcine colon tissue sample. (**E**) OCT B-scan of porcine colon (average of 10 consecutive frames, displayed in polar coordinates). (**F**) OCT 3D view of the porcine colon. The imaging experiments were performed using the 16 mm OCT-rectoscope. The covered area corresponded to approximately 50 × 10 mm^2^. The acquired frames exhibited a jitter of ± 4 A-scans but retained adequate phase stability for consecutive frame averaging (**B**, **E**). The densely sampled data sets were acquired within 2 s and consist of 4800 × 1334 × 1200 voxels, translating to a file size of 14.6 GB.

The RV1 probe was employed to capture images of the colon surface of a silicone colonoscopy training model (Olympus Colon-Model-CM-1) as shown in Fig. 2A. The laser bandwidth was set at 100 nm, the incident power at 18 mW acquiring 4800 A-Scans per rotation. To perform 3D imaging, the OCT rectoscope was manually pulled back while the silicone material was wrapped around the probe's circumference to ensure proper contact. A total of 1334 B-scans were acquired per volume and subsequently displayed in post-processing.

### Ex vivo porcine colon and histology

To facilitate our investigation into the efficacy of OCT imaging for intestinal tissue, we utilized freshly explanted porcine colon as mentioned above.

The RV1 probe was utilized for this phase of the study, where it was carefully inserted into the lumen of the porcine colon as shown in Fig. 2D. To achieve a thorough internal scan, we manually pulled back the probe by 1 cm at an approximate rate of 5 mm/sec. This procedure involved capturing 4800 A-scans per rotation and 1334 B-scans, utilizing a 108 nm spectral bandwidth, with an incident power of 21 mW on the sample.

After completing OCT imaging, our methodology for validating the imaging findings involved the precise excision of two tissue samples from the imaged colon sections. These excisions were performed with a scalpel, ensuring accuracy in correlating histological analysis with specific OCT-imaged locations. The tissue samples were then prepared for histological examination. Utilizing a 12-mm biopsy punch, we prepared the sections for fixation. This step is critical for preserving tissue morphology for detailed analysis. The prepared samples were pinned to cork supports and immersed in a 4% (w/v) phosphate-buffered paraformaldehyde solution, where they remained for 3 days at 6 °C to ensure thorough fixation. The fixed tissues were embedded in paraffin followed by subsequent sectioning into 5 µm slices. The slices were stained with haematoxylin and eosin (H&E) and evaluated using a Leitz–Leica orthoplan microscope.

### Post mortem human colon in-situ and histology

A body donor (82 years, male, 176 cm, 82 kg) was obtained from the body donation program (KS-2003) of the Institute of Anatomy, Christian-Albrechts-University, Kiel, Germany, as mentioned above. The body underwent fixation with an ethanol-glycerol- lysoformin solution through the femoral arteries, as previously described, to preserve normal tissue consistency^36^. The fixation protocol utilized a solution composed of 70% ethanol, 30% glycerol, and 0.3% lysoformin, with a total volume of approximately 0.3 l/kg body weight. Following fixation, the colon was thoroughly rinsed intraluminally with water and then ligated transabdominally at the rectosigmoid junction to prepare for imaging.

Imaging of the colon was conducted using standard flexible sigmoidoscopy, employing an Olympus colonoscope (CF-H190I) with a compatible endoscope processor (EVIS-Exera-III, CV-190-Plus, Olympus, Hamburg, Germany). Subsequently, OCT measurements were conducted with the RV2 probe, covering a range of 1–8 cm via manual pullback. This procedure involved capturing 4900 A-scans per rotation and up to 5120 B-scans, utilizing a 100 nm spectral bandwidth, with an output power of approximately 30 mW. The B-scans were visualized in real-time, in both Cartesian and polar coordinates, and were complemented with en-face and live 3D previews. To enhance the signal-to-noise ratio (SNR) and improve tissue structure visualization, a live averaging technique was applied, combining four consecutive frames to achieve a preview update rate of approximately 170 Hz.

After in-situ endoscopic imaging experiments, the sigmoid colon and rectum were excised and removed from the body for further analysis. This analysis was conducted with a benchtop scanning unit of the MHZ-OCT system, as explained and described in detail in the supplementary material (Fig.S2). Full-thickness tissue samples, including a rectal polyp from both the sigmoid colon and rectum, were then processed. The samples underwent dehydration, embedding in paraffin, sectioning into 5µm slices, and staining with Hematoxylin & Eosin (H&E) and Azan for histological examination. The stained sections were examined and documented using a BZ-X810 microscope (Keyence, Japan).

## Results

A systematic examination was performed to assess the potential of OCT for the diagnostic evaluation and phenotyping of rectal diseases, with an emphasis on applications that could be translated to in vivo settings. The initial phase of the study involved the utilization of a silicon colon model with the RV1 probe, aiming to establish baseline imaging benchmarks in a controlled, non-biological environment. This foundational work was imperative for gaining an initial understanding of the OCT rectoscope's performance. Subsequent phases included the application of OCT imaging to ex vivo porcine colon tissues, thereby bridging the gap between simplistic models and complex biological tissues. Encountering certain limitations with the RV1 probe in these more realistic settings necessitated the adoption of a second-generation probe, RV2, for further experimentation, which was applied to post-mortem human colon specimens in situ. This incremental approach, from basic models to tissues that approximate in vivo conditions, was designed to methodically confront and surmount initial technological and methodological hurdles, culminating in a demonstration of the OCT system’s refined diagnostic capabilities.

### Silicon colon model

For the preliminary testing of the prototype of the OCT rectoscope, profilometric imaging was conducted inside a silicon colon model (Fig. 2A). Although the silicon colon lacks different tissue structures, it does contain macroscopic silicone polyps for profilometric imaging. The OCT cross-sectional view (Fig. 2B) showed a prominently scattering surface layer with a uniform, non-scattering tissue layer underneath. A three-dimensional rendering of the manual pullback data (Fig. 2C) clearly depicted the folded surface structure of the colon model. A dark shadow of the motor wires was visible in the top part of the volume. The minimal distortion evident in the rendered volume indicated stable motor motion and a smooth manual pullback process.

### Ex vivo porcine colon

3D-OCT datasets were acquired from the excised porcine colon using identical OCT settings (Fig. 2D). With a high frame rate of ~ 667 Hz, the resulting OCT images exhibited a notable signal intensity. Capturing a generously sized FOV measuring 50 mm in circumference, the images provided substantial signal contrast throughout a penetration depth of roughly 1 mm. Each cross-sectional image was derived from an averaging process applied to 10 consecutive frames. Consequently, the entire colonic wall was clearly discernible in both the cross-sectional images (Fig. 2E) and the three-dimensional rendering (Fig. 2F).

**Figure 3:**
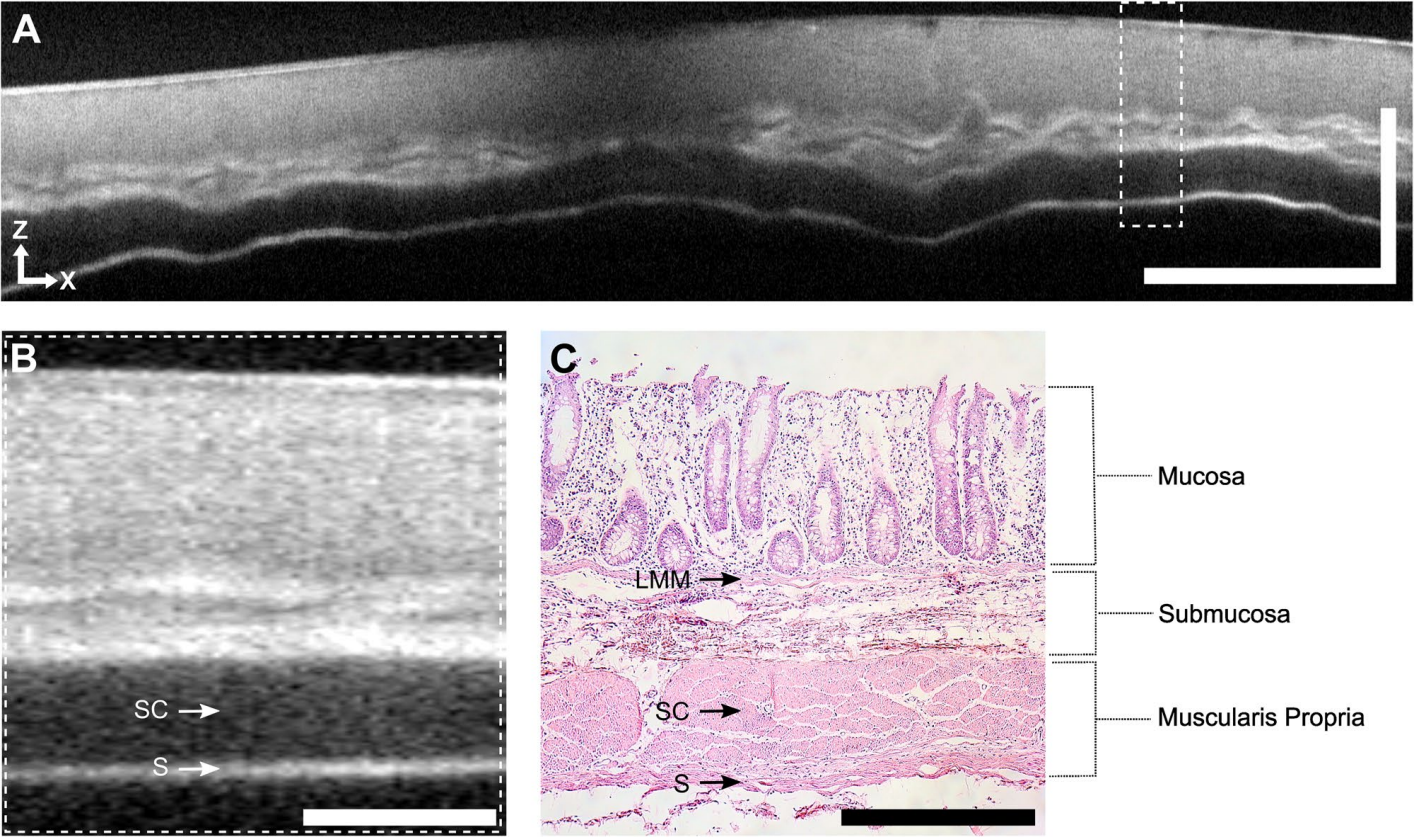
Endoscopic OCT and histology of porcine colon. (**A**) OCT B-scan of a porcine tissue sample (average of 10 consecutive frames and 2 A-scans, displayed in cartesian coordinates). Scale bar: x = 5 mm, z = 1 mm. (**B**) Higher magnification of the highlighted region (dotted rectangle) indicated in A. Scale bar: 500 μm (equal spacing for x and z). (**C**) Histology (H&E staining) of the tissue excised at the position indicated in A (dotted rectangle). Scale bar: 500 μm (equal spacing for x and z). Abbreviations: LMM, lamina muscularis mucosae; SC, stratum circulare; S, serosa.

Utilizing OCT, the mucosa appeared as a pale gray band, distinctly bordered by the lamina muscularis mucosae, while the submucosa was visualized as an inhomogeneous, light gray area. Furthermore, the muscularis propria manifested as a homogeneous dark gray band, succeeded by the serosa displayed as a narrow bright band. This observation indicated the comprehensive capture of the entire colonic wall by OCT endoscopy (Fig. 3A,B). Remarkably, the demarcation of the different colonic wall layers closely correlated with the respective H&E sections obtained from the porcine colon (Fig. 3C).

**Figure 4:**
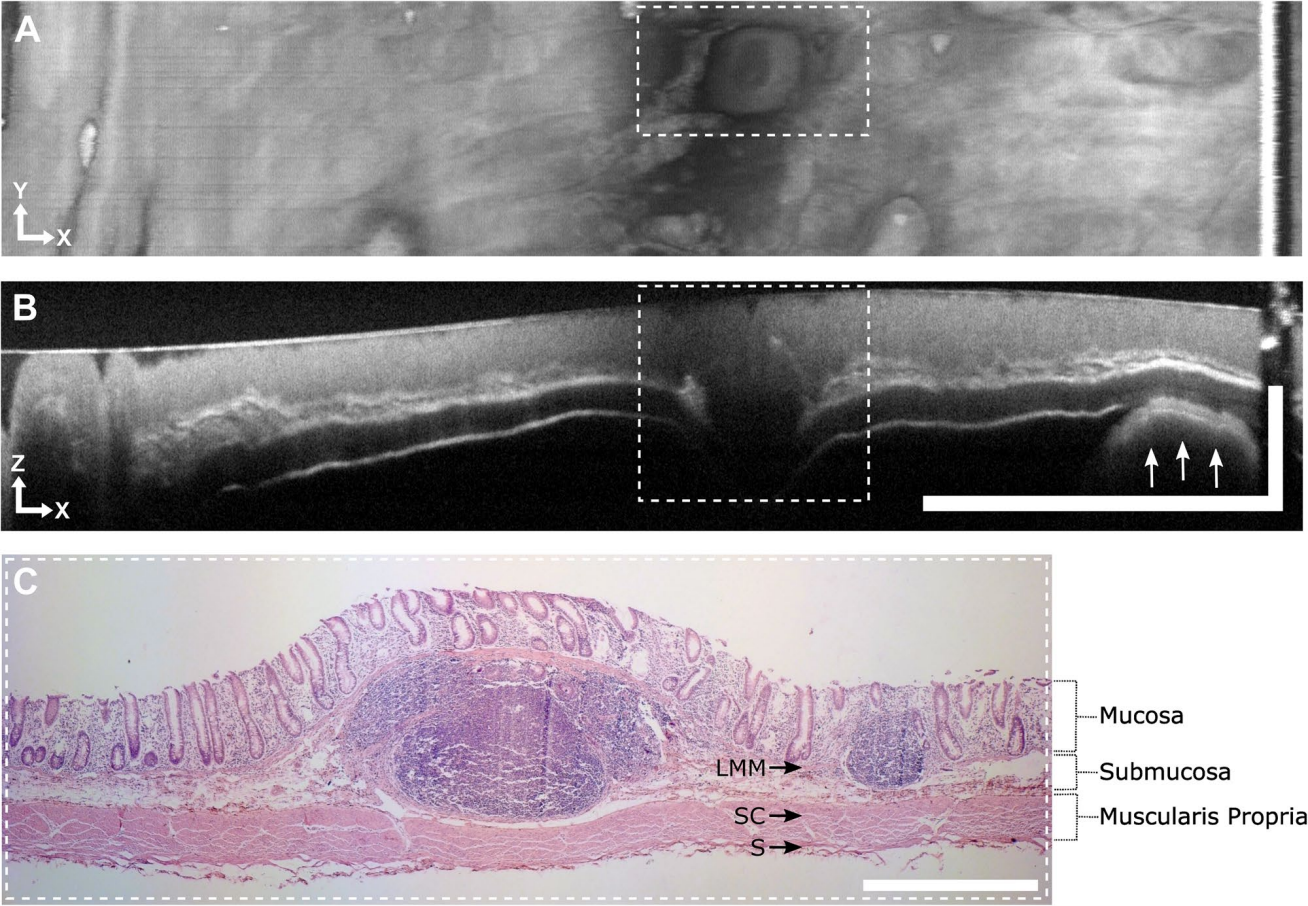
Gut associated lymphatic tissue in OCT endoscopy and histology of porcine colon. (**A**) OCT *en face* projection of 150 depth layers showing nodular lymphatic follicles (dashed rectangle). (**B**) OCT B-scan showing a lymphatic follicle (dashed rectangle) (average of 10 consecutive frames and 2 A-scans, displayed in cartesian coordinates). Arrows indicate the finger of the researcher. Scale bar: x = 10 mm, z = 1 mm. Motion artifacts visible in the *en face* view were relatively small during pull back. The jaggy shape of the motor wires resulted from the remaining synchronization error of motor and FDML tuning speed. (**C**) Histology (H&E staining) of the tissue excised at the position indicated in B (dashed rectangle). Scale bar: 1 mm (equal spacing for x and z). Abbreviations: LMM, lamina muscularis mucosa; SC, stratum circulare, S, serosa.

In a second colon sample, we identified gut-associated lymphatic tissue. During OCT examination, these lymphatic follicles presented as submucosal nodular structures with minimal scattering, distinctly discernible in both projections (Fig. 4A,B) and correlating well with the histological sections (Fig. 4C).

### Post mortem human colon in-situ

**Figure 5:**
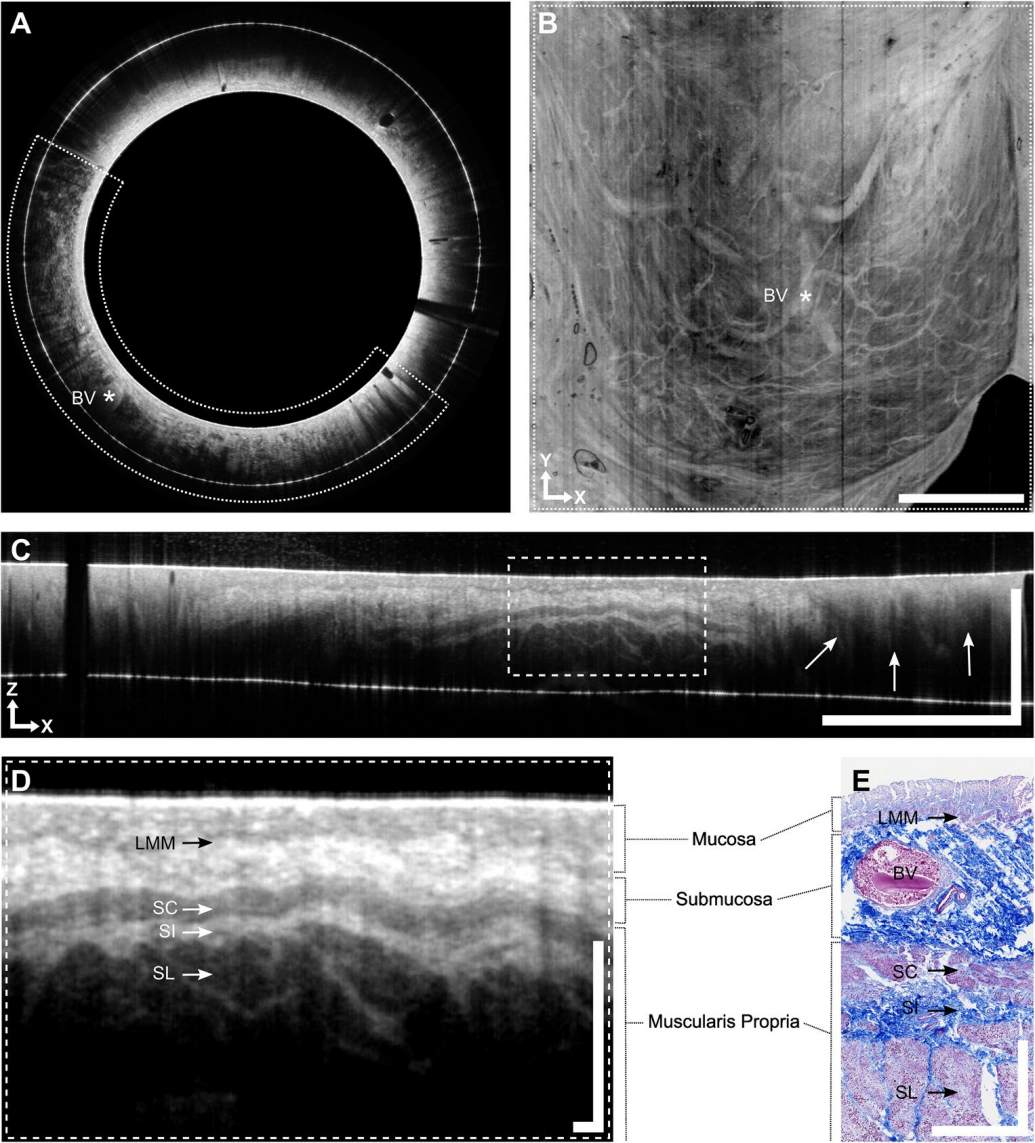
Endoscopic OCT and histology of post mortem human rectum in-situ. (**A**) OCT B-scan displaying vascular depth structure (large blood vessel marked with asterisk) (average of 10 consecutive frames, displayed in polar coordinates). Diameter of the probe: 25 mm. (**B**) OCT *en face* projection of all depth layers displaying submuscular vascular networks (corresponding blood vessel to A marked with asterisk). The displayed area corresponds to the region indicated in A (dotted semicircle). Scale bar: 10 mm (equal spacing in x and y). (**A**, **B**) The original dataset displayed consists of 4900 × 5120 A-scans with 1200 samples per A-scan, resulting in a total data set size of 44 GB. The large dataset was acquired within 7.7 s and streamed instantaneously to storage disk. A copy of the data in the RAM was used for live display during the procedure. The high imaging speed and preview update rates enabled live averaging of consecutive frames resulting in higher signal to noise ratios and thus improved image preview quality. (**C**) OCT B-scan displaying the polyp (arrows) (average of 10 consecutive frames and 2 A-scans, displayed in cartesian coordinates). Scale bar: x = 10 mm, z = 1 mm. The white line (in lower third of the image) corresponds to the inner PMMA surface of the imaging window, which is flipped into the OCT image. Due to the 2 mm PMMA wall thickness this artifact does not interfere with the OCT signal with a penetration depth of approx. 1 mm. D) Higher magnification of the highlighted region indicated in C (dotted rectangle). Scale bar: 500 μm × 500 μm. (**E**) Histology (Azan staining) of the tissue excised at similar position compared to D. Scale bar: 500 μm × 500 μm. Abbreviations: BV, blood vessel; LMM, lamina muscularis mucosae; RAM, random-access memory; SC, stratum circulare; SI, stratum intermusculare; SL, stratum longitudinale.

In a next step, we conducted in situ imaging of a body donor`s rectum to assess the clinical efficacy of the OCT rectoscope. Conventional recto-sigmoidoscopy revealed that the ethanol-glycerol-lysoformin fixation method effectively preserved the true-to-life anatomy and tissue consistency of the colon. This fixation technique provided optimal conditions for rectal examinations, owing to the tissue's flexibility, and also maintained the integrity of the mucosa. In this particular body donor, a relatively narrow rectum was observed, with three polyps identified in the rectosigmoid region (Fig. S1). The 25 mm diameter rectoscope was introduced into the rectum and allowed almost circumferential contact with the mucosa in its deflated state. Based on the exceptionally high imaging speed, an extensive examination area with a circumference of 83 mm and high signal contrast was achieved (Fig. 5).

In the cross-sectional view (Fig. 5A), several blood vessels of the submucosal vascular plexus were detected up to a penetration depth of approximately 1mm. The transition from the B-scan in polar coordinates (Fig. 5A) to the *en face* projection (Fig. 5B) illuminated the nuances of the vascular network. Barely any motion artifacts were visible in the *en face* view (Fig. 5B), indicating a stable manual pullback procedure of approximately 10 mm/s. The high-speed performance and real-time display of the data are shown in the screen recordings (Video S2). OCT rectoscopy itself clearly depicted a polyp (Fig. 5C,D). The polyp exhibited a low signal intensity contrasting with the higher signal intensity of the normal adjacent mucosa (Fig. 5C). An OCT *en face* representation of the dataset and histology of the polyp is visualized in Fig. S1. Comparable to the porcine colon, we were able to clearly identify the mucosa, submucosa, and muscularis propria. Identification of the lamina muscularis mucosae allowed exact delineation of the mucosa from the underlying submucosal layer. Within the muscularis propria, the circular and longitudinal muscle layers were displayed as dark gray bands, whereas the intermuscular plane appeared as a narrow white band (Fig. 5D). The morphological features correlated well with the histology taken from the same region (Fig. 5E).

To evaluate the highest resolution of our OCT setting with potential visualization of colonic crypts in further detail, we performed a benchtop MHZ-OCT scan using the removed rectum of the body donor. Respective details are described in the supplemental material (Fig. S2).

## Discussion and conclusion

High-resolution visualization of the mucosal surface and differentiation of the various layers of the rectal wall are crucial for the accurate initial diagnosis and assessment of treatment efficacy in rectal malignancies and chronic inflammatory conditions. While traditional high-resolution white-light endoscopy provides valuable information about surface structure, it has limitations in probing beyond the superficial mucosal layer. EUS complements this by imaging deeper tissue structures delineating the transmural wall structure^5^ and quantifying inflammation levels but struggles to distinguish fine structures. On the other hand, CLE offers detailed microscopic visualization of capillary and crypt structures but lacks tissue penetration and field of view^6^. Therefore, despite the distinct advantages of each technique, the aspiration for 3D imaging of the entire rectal wall in real-time or within a clinically feasible timeframe remains challenging. Our study overcomes these limitations by offering enhanced imaging capabilities, aligning with clinical needs for swift and accurate diagnostics.

To address the challenges of comprehensive rectal imaging, our study introduces an "all-in-one" MHz-OCT rectoscope, designed for 3D imaging of the entire rectum with high resolution and adequate penetration depth, operating at an A-scan rate above 3.2 MHz. Our development process led to the creation of two circumferential scanning OCT rectoscopes with outer diameters of 16 mm (RV1) and 25 mm (RV2). We initiated our testing process on a silicone colon model, laying the groundwork for subsequent trials on a porcine colon using the RV1 probe. Building upon our findings and acknowledging the limitations of RV1, we proceeded to develop RV2. This updated version features enhanced OCT triggering electronics, significantly reducing jitter to less than ± 4 A-scans compared to the initial ± 40 A-scans of RV1. Subsequently, RV2 was utilized for the analysis of post mortem human colon in situ. Additionally, for rectoscopy procedures, we employed an ethanol-glycerol-lysoformin fixation method. This approach not only preserved the true-to-life anatomy and tissue consistency of the colon but also resulted in remarkably high OCT image quality. Our observations suggest that this fixation method maintains tissue contrast better than standard formalin fixation. This approach not only confirmed the rectoscope's clinical relevance but also highlighted its potential to significantly advance gastrointestinal imaging by providing precise, real-time 3D visualization of rectal diseases.

Achieving an axial resolution of up to 8 µm, our enhanced endoscope enables real-time differentiation of distinct colonic tissue layers at an acquisition speed previously unattainable. Its optical configuration, boasting an imaging range of 5 mm and a spot size of 35 µm in air (27 µm in tissue), has proven more than sufficient for visualizing the intricate folding architecture of the colon and its 3D reconstruction. This capability is crucial for clearly differentiating various tissue layers, lymphatic tissues, blood vessels, and endoluminal polyps. Notably, the capacity to delineate the lamina muscularis mucosae from the submucosal tissue is particularly important for diagnosing neoplastic and inflammatory diseases, which can be distinctly identified using our MHz-OCT endoscope. Despite these advancements, it is recognized that our system currently lacks the resolution necessary to resolve crypt structures. This limitation primarily stems from the use of B-scan averaging during measurements and inherent resolution constraints. However, it is worth noting that crypt structures are clearly observable in comparative MHz-OCT measurements conducted on excised human colon specimens with a benchtop system, which features a lateral resolution of 20 µm in air. This highlights a trade-off inherent to our system's design, balancing high imaging speed against the granularity of detail, which nonetheless enables the capture of large *en face* OCT images spanning several square centimeters with remarkable contrast. Our dataset reveals intricate morphological details with minimal motion artifacts, attributable to the precision of the manual pullback technique employed. The FOV provided by our system is instrumental in identifying a diverse range of tissue structures that may not be easily observed in individual cross-sectional views, including detailed vascular patterns. This extensive coverage is instrumental in enhancing diagnostic capabilities, allowing for the identification and characterization of a wide array of tissue anomalies and pathologies. This reinforces the value of our MHz-OCT system as a significant advancement in the field of gastrointestinal diagnostics, offering a pragmatic balance between speed and resolution that aligns with clinical needs.

In general, OCT's potential for endoscopic imaging is vast. Evaluating morphological features, such as vascular patterns, can provide insights ranging from vascular anomalies to early indicators of neoplastic and inflammatory disorders. Moreover, extensive FOV aids in recognizing irregular tissue features, making it a valuable tool for AI-based automated feature recognition. Our MHz-OCT system's utilization could further enhance the AI-based detection of neoplastic precursors, marking a significant advancement beyond previous studies' capabilities. Upon comparing the imaging capabilities of our endoscope with those reported in the existing literature, it becomes apparent that our system achieves a resolution somewhat lower than that of prior studies dedicated to high-resolution imaging^15,16^. Such studies, notably including the work by Zeng et al. were proficient in delineating crypt structures within the mucosa, crucial for the automated detection of colorectal cancer utilizing slower OCT systems endowed with higher resolutions^20^. Despite this disparity, our endoscope excels in facilitating whole colon imaging. It provides a significantly extended FOV and an augmented imaging speed, with A-scan rates surpassing 3.2 MHz, far exceeding the sub-100 kHz rates observed in earlier studies^16^. The resolution of our endoscope, while currently identified as a limiting factor, is slated for enhancement in subsequent iterations of our research.

Previous studies, such as those conducted by Adler et al., primarily utilized probe-based systems for initial in vivo imaging experiments in humans^16^. However, they encountered limitations due to insufficient imaging speed, which hindered the translation into durable clinical human applications. Our study bridges this gap, showcasing an ultrafast OCT technology capable of precise visualization and differentiation of intestinal layers in conditions like rectal cancer and IBD, directly overcoming the constraints highlighted by prior research. The efficacy of EUS in assessing infiltration depths in rectal cancer contextually underscores the importance of our findings^37^, which align with Luo et al.'s comprehensive analysis on colorectal specimens. This analysis demonstrated our endoscope's capability to distinguish between normal and cancerous tissues accurately^38^, enhancing the clinical utility of OCT technology.

Achieving precise in vivo imaging in IBD is essential for accurately assessing disease states from onset through treatment. OCT, with its ability to replace random biopsies with targeted ones, exemplifies significant advancements in real-time visualization of colonic tissue layers, potentially distinguishing Crohn’s disease (CD) from ulcerative colitis (UC). This is supported by Ding et al., who observed meaningful correlations between histological inflammation and mucosal as well as submucosal wall thickness assessed by OCT in a dextran sulfate sodium mouse colitis model ex vivo^39^. Furthermore, Shen et al.’s^23^ study, which involved 40 CD patients and 30 UC patients, revealed that a disruptive layered structure was present in 90.0% of CD patients as opposed to only 16.7% of UC patients, as identified through OCT imaging. These findings, in conjunction with our group's previous demonstrations using EUS^5^, not only reinforce the clinical applicability of our MHz-OCT system but also highlight its potential to enhance diagnostic precision and differentiation between IBD entities. Our study thus contributes to the promising field of gastroenterological diagnostics, showcasing the critical role of advanced OCT technologies in improving the accuracy and efficiency of IBD management.

In UC, detecting dysplasia-associated lesions and benign inflammatory pseudopolyps, characterized by preserved stratification of colonic wall layers, is crucial for effective rectal cancer screening. Our previous study's success in identifying polyps through OCT imaging supports the modality's potential as a non-invasive optical biopsy tool, enhancing patient safety with real-time imaging^22,26^. This capability, combined with the potential of Doppler OCT in mice^11^ imaging applications, as demonstrated by Welge and Barton in their utilization of in vivo endoscopy, distinguishes adenoma from healthy tissue based on perfusion properties. Similarly, dynamic OCT (dOCT), which depends on cellular motion, enables the visualization of additional functional information^40,41^. Furthermore, the anticipated integration of dynamic optical contrast into the OCT-rectoscope is expected to expand its applicability, potentially including the assessment of gastrointestinal involvement in neurodegenerative and neuroinflammatory diseases.

Our findings represent a notable stride towards bridging the gap between OCT as a purely research-focused tool and its practical application in a clinical context. This is particularly relevant considering the substantial number of diagnoses for the diseases in question that can be conclusively determined within the rectum. The design of our rigid OCT-rectoscope, with its larger diameter, not only leverages cost-effectiveness but also enables a wide FOV, crucial for diagnosing rectal pathologies. There's an ambitious plan to extend its applicability further. We aim to adapt the optical scanning mechanism for use with a flexible endoscope or colon capsule^42^, potentially revolutionizing comprehensive colon scans with densely sampled 20 µm pixel sizes within a 45-s timeframe. Our commitment to refining the OCT-rectoscope's functionality for spectral zooming and swift transitioning between low-resolution and long-range imaging modes promises substantial improvements in pathology visualization. It's important to note that our current dataset, derived from post-mortem models, necessitates further research to validate the system’s effectiveness in live clinical settings, requiring a broader in vivo sample size for thorough validation.

In summary, given its non-invasive nature and capability to provide immediate diagnostic results, OCT emerges as an inevitable tool in advancing our understanding and management of rectal diseases. We anticipate that it may considerably impact the future of gastrointestinal disease diagnosis and treatment, offering a powerful, non-invasive method for instantaneously distinguishing between healthy and pathological conditions.

### Supplementary Information

Supplementary Information 1.

Supplementary Figure S1.

Supplementary Figure S2.

Supplementary Figure S3.

Supplementary Figure S4.

Supplementary Video 1.

Supplementary Video 2.

## Data Availability

The datasets generated during the current study are recorded at the BMO Luebeck, University Luebeck, Luebeck, Germany. The datasets used and/or analysed during the current study are available from the corresponding author on reasonable request.

## Abbreviations

3D: Three dimension
NA: Numerical aperture
BEMF: Back electromotive force
CD: Crohn’s disease
CLE: Confocal laser endomicroscopy
cm: Centimeter
CNN: Convolutional neural network
DC: Direct current
dOCT: Dynamic optical coherence tomography
EUS: Endoscopic ultrasound
FDML: Fourier domain mode locking
Fig.: Figure
FOV: Field of view
fps: Frames per second
GiB: Gibibyte
GPU: Graphical processing unit
GS/s: Giga samples per second
H&E: Haematoxylin and eosin
IBD: Inflammatory bowel disease
l: Litre
kg: Kilogram
LURO: Luebeck University real-time OCT
MHz: Megahertz
mm: Millimetre
mrad: Milliradian
µm: Micrometer
mW: Milliwatt
NaCl: Sodium chloride
nm: Nanometer
NURD: Non-uniform rotational distortion
OCT: Optical coherence tomography
PMMA: Polymethyl methacrylate
RAM: Random-access memory
Rpm: Revolutions per minute
s: Second
SD: Spectral domain
SNR: Signal to noise ratio
UC: Ulcerative colitis
UHR: Ultrahigh-resolution
VLE: Volumetric laser endomicroscopy
Vol: Volume
w/v: Weight per volume
