# Supplementary material for: High-resolution rectoscopy using MHz optical coherence tomography: a step towards real time 3D endoscopy
Source: Sci Rep. 2024 Feb 26;14:4672. doi: 10.1038/s41598-024-55338-5 (PMC10897148; doi:10.1038/s41598-024-55338-5)
Supplement: Supplementary file 1 — Supplementary Information 1. [file 41598_2024_55338_MOESM1_ESM.docx]

High-resolution rectoscopy using MHz optical coherence tomography - a step towards real time 3D endoscopy.

Berenice Schulte#^1^, Madita Göb#^2^, Awanish Pratap Singh^2^, Simon Lotz^2^, Wolfgang Draxinger^2^, Marvin Heimke^3^, Mario pieper^4,5^, Tillmann Heinze^3^, Thilo Wedel^3^, Maik Rahlves^2^, Robert Huber^2^, Mark Ellrichmann*^1^

*^1^ Interdisciplinary Endoscopy, Medical Department I, University Hospital Schleswig-Holstein, Campus Kiel, Kiel, Germany*

^2^ Institute of Biomedical Optics, University of Luebeck, Luebeck, Germany

*^3^ Institute of Anatomy, Center of Clinical Anatomy, Christian-Albrechts University Kiel, Kiel, Germany*

*^4^ Institute of Anatomy, University of Luebeck, Luebeck, Germany*

*^5^ Airway Research Center North (ARCN), German Center for Lung Research (DZL), Lübeck, Germany*

# B. Schulte and M. Göb contributed equally to this work

**Supplemental Material**

**Appendix**

LURO data handling and real-time processing

In order to acquire TiB-scale data required for future vision of full colon imaging and to achieve real-time live low latency display for interactive positioning and aiming mode, we developed a specific software solution. This real-time processing and visualization of OCT data is based on our custom in-house software for online GPU accelerated OCT processing in real-time “LURO” (Luebeck University Real-time OCT) navigation[1,2]. Data acquisition is executed in “triggered streaming” mode, where for every B-scan – a single rotation – initiated by FDML[3] fundamental period synchronized trigger signal a fixed number of samples is captured as a cohesive data segmented and transferred into the computer system’s main memory. The digitizer and the GPUs are hosted in a computer system using a Xeon W CPU (Intel, Xeon W-2245, USA), which has 8 cores that support 16 concurrent threads, running at a 3.9 GHz base clock. The computer system offers 256 GiB of RAM, of which 216 GiB were reserved as a FIFO buffer between data acquisition, processing and permanent storage. For each captured segment in flight the LURO software schedules concurrent processing of the data across all the GPUs available in the system, and in case a capture to storage is in progress also writes out the data in parallel. If the memory on the GPU is large enough to hold a fully processed C-scan, the processed data is immediately visualized, also online and in real-time, as either B-scan aligned cross sections in Cartesian or polar mapping, Cartesian C-scan aligned en face projection, or full resolution volume rendering. The computer system that was used for this study operates on single GeForce RTX 3070 GPU (Nvidia, USA), which boasts sufficient computational power, to single handedly execute all these operations. Should the extents of a single C-scan result in processed images that exceed the capacity of the GPU’s memory reserved for visualization, a sparse mode is available, for which only every N-th bunch of A-scans inside a FDML fundamental sweep are processed for immediate visualization; the sparsity divider N will then be chosen so that the range of a whole C-scan will fit into GPU memory for preview. Thus, the size of a single scan is limited by the available system memory and the constraints of the data acquisition system. In the “triggered streaming” mode, the ATS9373 card is limited to a segment size of 20×2^20^ samples per trigger event; with 1200 samples per A-scan this amounts to a maximum width of ~17.4k A-Scans per trigger event. Wider B-scans can be achieved by generating intra triggers. However, at every intra-trigger location, a gap of a single FDML fundamental period is introduced, caused by trigger re-arm and re-alignment with the FDML fundamental frequency. For storage there’s a M.2 form factor NVMe solid state disk (SSD; Samsung, 980 Pro, South Korea) with a 2 TiB capacity and sustained write speeds of up to 2 GiB/s. In combination with the 216 GiB of system RAM used as FIFO buffer, up to 45 s of 4D data can be captured, amounting to about 280 GiB of raw OCT interference fringe data, for later, offline post-processing, with the possibility to also apply advanced algorithms that go beyond the capability of the real-time software. This performance is already sufficient to acquire the approximately 250 GiB OCT data for a complete 3D volume of the entire colon using a spot size of 30-40 µm. For higher resolution imaging, the system capability can easily be extended.

Detailed postmortem human colon tissue analysis

OCT rectoscope imaging experiments were conducted in postmortem human colon *in situ* in order to mimic a true-to-life colonoscopy scenario. The body donor was fixed using an ethanol-glycerol-lysoformin fixation method, which in contrast to commonly used formalin fixation method is supposed to preserve soft tissue consistency[4,5].

In order to evaluate the comparability of the mock-up colonoscopy and fixation method with imaging conditions in living patients, we compared two standard colonosocopy images, shown in Figure S1. Figure S1A shows a polyp in the rectal lumen of the body donor (6 cm ab ano). Figure S1B depicts a similar sized polyp of a living patient. Both were acquired using an Olympus colonoscope (CF-H190I) with compatible endoscopy processor (EVIS Exera III, CV-190 Plus, Olympus, Hamburg, Germany). Compared to the patient´s colon, the post mortem colon appeared paler and brownish due to the absence of blood flow and post mortal biological tissue alterations. Nevertheless, the macroscopic observations revealed that the ethanol-glycerol-lysoformin fixation method effectively preserved the true-to-life anatomy and maintained integrity of the mucosa.

**Benchtop MHZ-OCT scan unit**

After in situ endoscopic imaging experiments, the respective segment of the sigmoid colon and the rectum including the polyp were excised and opened by longitudinal incision for imaging procedures using the standard benchtop scanning unit of the MHz OCT system.

The primary rationale for employing the benchtop MHZ-OCT scan unit was to compare the accuracy and performance of our novel rectoscope with that of a benchmark OCT system, known for it`s stability and absence of jitter and image artifacts caused by manual pull-back. Our rectoscope was designed to provide high-quality in vivo imaging with minimal disruption, a critical advancement for clinical applications. By generating higher-resolution images using the benchtop MHZ-OCT unit, we aimed to demonstrate that our rectoscope could achieve a level of resolution in vivo that was comparable to the benchmark benchtop OCT scan unit system, particularly in the detailed visualization of rectal crypt architecture. This comparison is vital for establishing the clinical relevance of our rectoscope as a reliable tool for gastrointestinal imaging.

The applied benchtop MHZ-OCT scan unit consists of a pair of galvanometer mirror scanners (dynAXIS 421, Scanlab GmbH, Germany) operated in an unidirectional scanning mode and simple 50 mm scan lens for telecentric raster scanning with a lateral resolution of 18 µm. The bandwidth of the FDML laser was set to 100 nm, which results in an axial resolution of 8 µm in tissue. As shown in Figure S2A, different regions of interest were acquired, comprising 2048×2048 A-scans, equivalent in size to 7.5×7.5 mm². Figure S2 B illustrates the previously examined polyp as 3D rendering. As visualized, OCT is capable of assessing the topography of the polyp, which is an essential feature for automatic polyp detection. Moreover, a visually examined healthy colon tissue area next to the polyp was examined (Fig. S2 C-D). Both, the *en face* representation (Fig. S2C) and cross-sectional view of the OCT dataset (Fig. S2D) reveal that the OCT benchtop scanning unit is capable of resolving crypts (indicated with white arrows). This finding indicates, that slightly increased resolving power of the OCT-rectoscope will possibly give access to visualize the crypt pattern in the colon, which is crucial for future developments considering automatic, AI-based pattern recognition[6].

Subsequent to the benchtop OCT analysis, full-thickness tissue samples were taken from both the sigmoid and rectal walls, along with the rectal polyp. The samples were dehydrated, embedded in paraffin wax, sectioned to a thickness of 5 µm, subjected to hematoxylin-eosin and azan staining, and then examined and documented using a BZ-X810 microscope (Keyence, Japan).

Figure S3 shows the comparison of the OCT-rectoscope images of the polyp in situ with the histology obtained from the tissue sample. The OCT *en face* imaging allowed clear delineation of the boundaries of the polyp (Fig. S3 A). In the OCT cross-sectional view the normal layering of the rectal wall is sharply interrupted and dissolved at the area of the polyp (Fig. S3 B). Histologic examination confirmed the presence of an hyperplastic polyp next to the normal histologic architecture of the rectal wall (Fig. S3 C).

References

1. Draxinger W, Theisen-Kunde D, Schuetz L, et al. Microscope integrated real time high density 4D MHz-OCT in neurosurgery: a depth and tissue resolving visual contrast channel and the challenge of fused presentation. In: ProcSPIE [Internet]. 2023. Available at: https://doi.org/10.1117/12.2670953

2. Strenge P, Lange B, Draxinger W, et al. Demarcation of brain and tumor tissue with optical coherence tomography using prior neural networks. In: ProcSPIE [Internet]. 2023. Available at: https://doi.org/10.1117/12.2670907

3. Huber R, Wojtkowski M, Fujimoto JG. Fourier Domain Mode Locking (FDML): A new laser operating regime and applications for optical coherence tomography. Opt Express. 2006; 14: 3225–37.

4. Wedel T, Ackermann J, Hagedorn H, Mettler L, Maass N, Alkatout I. Educational training in laparoscopic gynecological surgery based on ethanol-glycerol-lysoformin-preserved body donors. Ann Anat = Anat Anzeiger Off organ Anat Gesellschaft. 2019; 221: 157–64.

5. Ackermann J, Wedel T, Hagedorn H, et al. Establishment and evaluation of a training course in advanced laparoscopic surgery based on human body donors embalmed by ethanol-glycerol-lysoformin fixation. Surg Endosc. 2021; 35: 1385–94.

6. Zeng Y, Xu S, Chapman WCJ, et al. Real-time colorectal cancer diagnosis using PR-OCT with deep learning. Theranostics. 2020; 10: 2587–96.

**Supplementary Figures**

Figure S1: Graphical Abstract; High-resolution rectoscopy using MHz optical coherence tomography

achieves detailed images of the rectal wall layers and endoluminal polyps, is suitable for real-time

imaging during endoscopy and facilitates a 3D reconstruction.

**Figure S2:** Endoscopic images of a body donor and a patient A) Rectal lumen of a body donor (ethanol-glycerol-lysoformin fixation) displaying a polyp at 6 cm ab ano. B) Corresponding image of a patient (in vivo) with a similar sized rectal polyp.

F**igure S3:** Benchtop scanning OCT of excised human colon tissue post mortem. A) Excised rectum with polyp (dotted rectangle) and normal tissue area (dashed rectangle). B) 3D OCT rendering of the polyp scanned at the position indicated by the dotted rectangle in A. Scanning FOV: 7.5 x 7.5 mm². C) OCT *en face* projection of all depth layers of normal tissue scanned at the position indicated by the dashed rectangle in A. Scanning FOV: 7.5 x 7.5 mm². D) OCT B-scan corresponding to the OCT en face image at the position indicated by the dashed line in C (average of 10 consecutive frames and 2 A-scans, displayed in cartesian coordinates). Crypt structure (arrows) is discernbile. Scale bar: x = 1 mm, z = 1 mm; Zoom scale bar: x = 0.2 mm, z = 0.2 mm. E) Histology (Azan staining) of the excised tissue showing crypt structures.

F**igure S4:** Endoscopic OCT and histology of postmortem human colon in situ with polyp. A) OCT en face projection of ten consecutive depth slices at the center depth of B. Arrows indicate outline of polyp. B) OCT B-scan displaying the polyp (arrows) (average of 10 consecutive frames and 2 A-scans, displayed in cartesian coordinates). The position of the B-scan in the dataset is indicated by a dashed line in the en face image in B. The lower white line corresponds to the upper PMMA surface of the imaging window, which is flipped into the OCT image. Scale bar: x = 10 mm, z = 1 mm. C) Histology (Azan staining) of the excised tissue. The polyp protrudes into the rectal lumen. The stratum intermusculare between the circular and longitudinal muslce layer is artificially widened due to tissue processing (artifact). Scale bar: 1 mm. Abbreviations: SC, stratum circulare; SL, stratum longitudinale.

**Supplementary Videos**

**Supp. Video 1:** 3D-rendering of a rectal polyp obtained by benchtop MHZ-OCT scan within the removed rectum of the body donor.

**Supp. Video 2:** Fly through in Y- and Z-axis obtained by obtained by benchtop MHZ-OCT scan within the removed rectum of the body donor.
