# Supplementary figures and images for: High-resolution rectoscopy using MHz optical coherence tomography: a step towards real time 3D endoscopy
Source: Sci Rep. 2024 Feb 26;14:4672. doi: 10.1038/s41598-024-55338-5 (PMC10897148; doi:10.1038/s41598-024-55338-5)

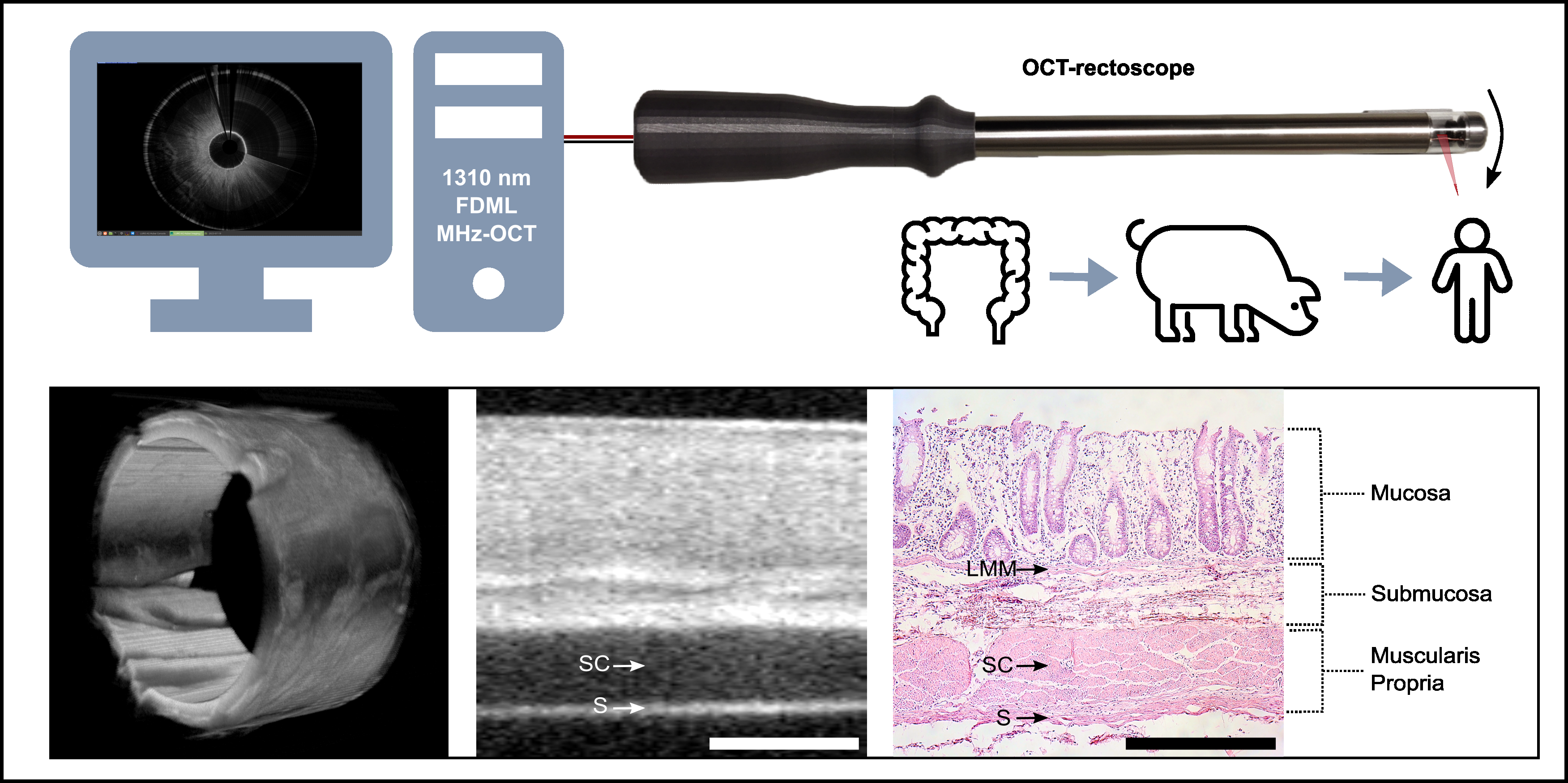

Supplement: Supplementary file 2 — Supplementary Figure S1. [file 41598_2024_55338_MOESM2_ESM.tiff]

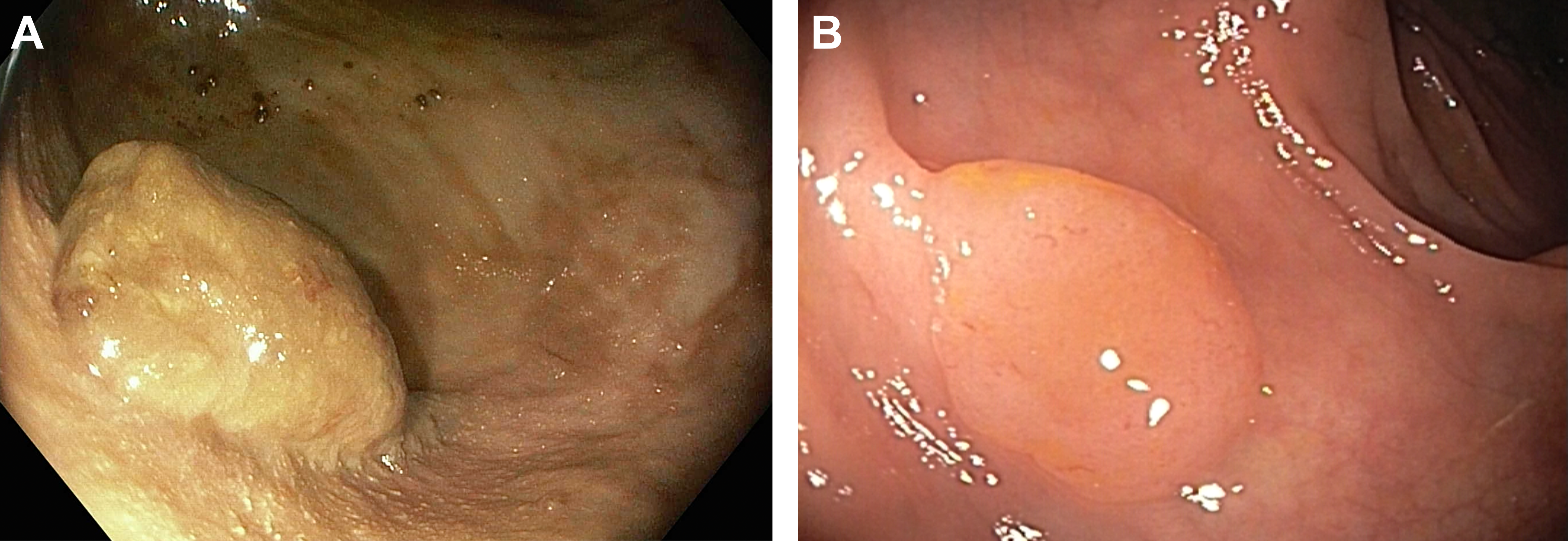

Supplement: Supplementary file 3 — Supplementary Figure S2. [file 41598_2024_55338_MOESM3_ESM.tiff]

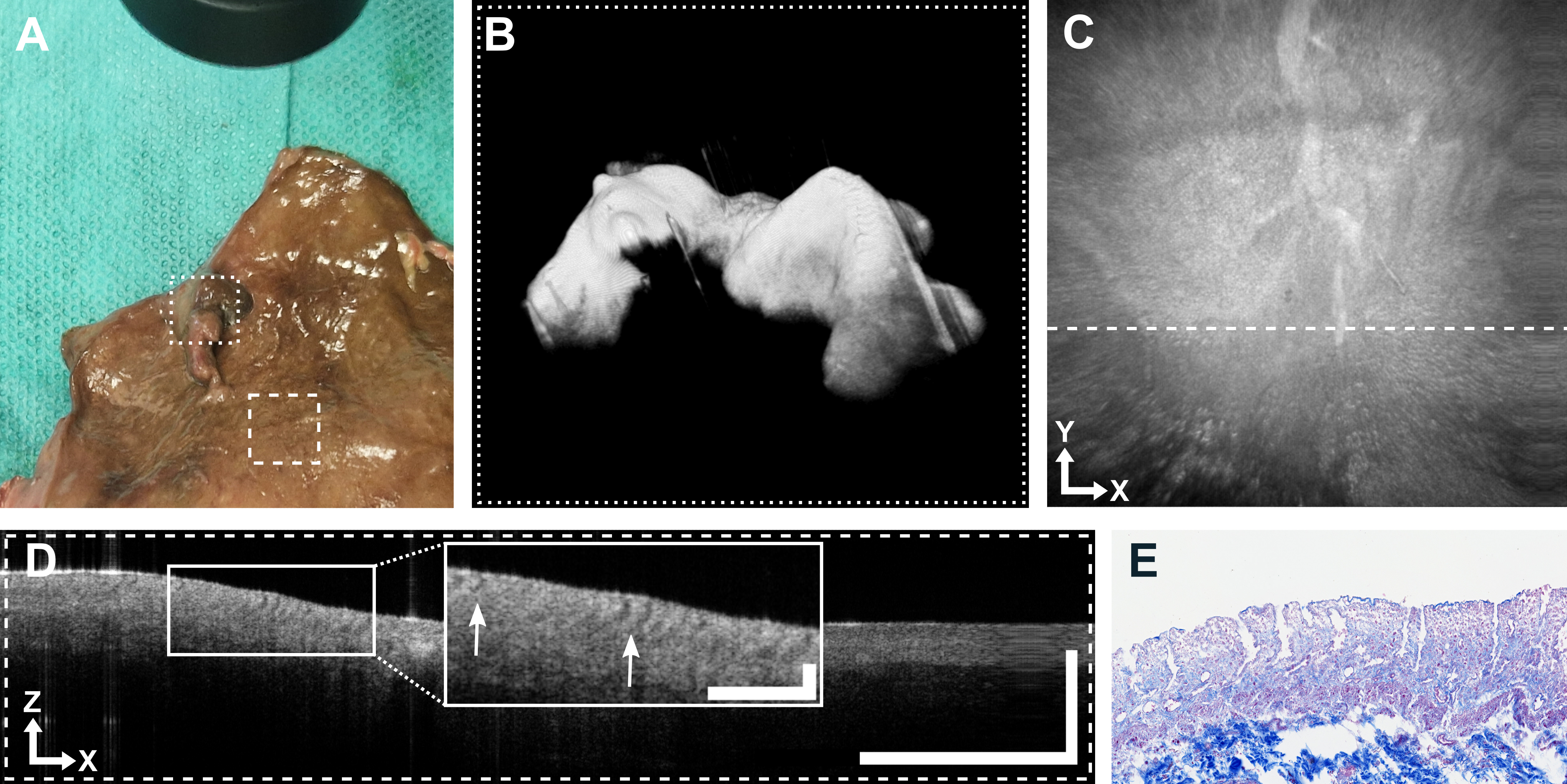

Supplement: Supplementary file 4 — Supplementary Figure S3. [file 41598_2024_55338_MOESM4_ESM.tiff]

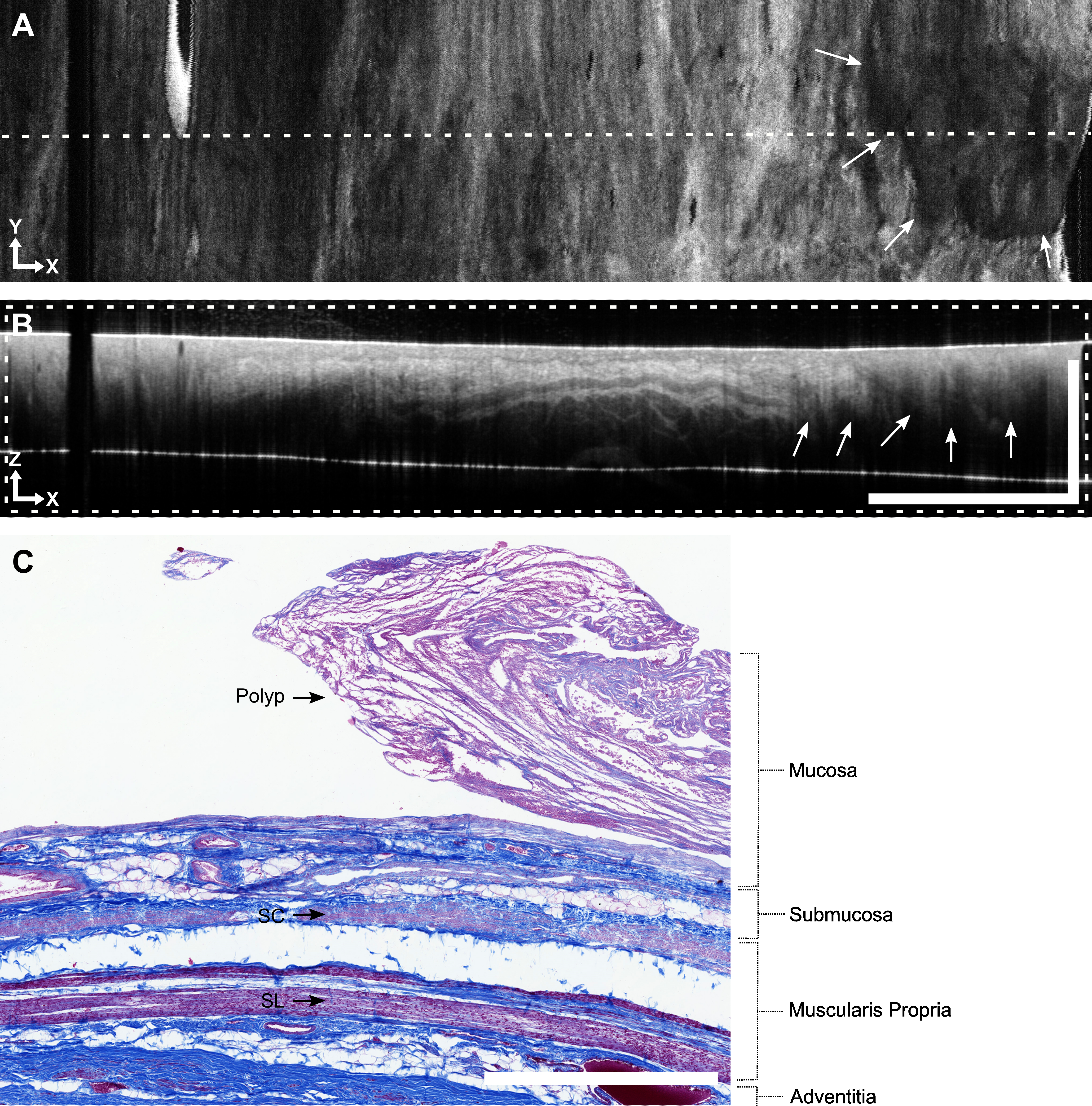

Supplement: Supplementary file 5 — Supplementary Figure S4. [file 41598_2024_55338_MOESM5_ESM.tiff]
